# Green Synthesis of Zirconium Dioxide Nanoparticles Using *Chlorella vulgaris* Extracts with High Antibacterial and Antifungal Properties

**DOI:** 10.3390/ijms27177803

**Published:** 2026-08-31

**Authors:** Beyzanur Erk, Bengü Ergüden

**Affiliations:** 1 Department of Biomedical Engineering, İstinye University, 34396 İstanbul, Türkiye; beyzanur.erk@istinye.edu.tr; 2 Department of Bioengineering, Gebze Technical University, 41400 Kocaeli, Türkiye

**Keywords:** *Chlorella vulgaris*, zirconium dioxide, ZrO_2_, nanoparticles, green synthesis, antimicrobial nanomaterials, sustainability

## Abstract

Green synthesis has emerged as a sustainable alternative to conventional nanoparticle production by utilizing biological resources as reducing and stabilizing agents. In this study, extracts of *Chlorella vulgaris* cultured at different incubation temperatures and light periods were evaluated for their total phenolic content, total flavonoid content, and antioxidant capacity to identify the most suitable extract for the green synthesis of zirconium dioxide (ZrO_2_) nanoparticles. The extract prepared at 25 °C under a 12 h light/12 h dark photoperiod exhibited the highest antioxidant activity and flavonoid content and was selected for nanoparticle synthesis. ZrO_2_ nanoparticles were successfully synthesized using a green approach and characterized by dynamic light scattering (DLS), Fourier-transform infrared spectroscopy (FTIR), scanning electron microscopy (SEM) and X-Ray diffraction (XRD). DLS analysis revealed hydrodynamic particle sizes ranging from 419 to 570 nm with polydispersity index values between 0.27 and 0.44. FTIR analysis confirmed the formation of ZrO_2_ through characteristic Zr–O and Zr–O–Zr vibrational bands, while SEM images demonstrated predominantly spherical particles with a heterogeneous size distribution. The synthesized nanoparticles retained considerable phenolic and flavonoid contents and exhibited enhanced antioxidant activity compared with the corresponding algal extracts. Furthermore, the nanoparticles demonstrated broad-spectrum antibacterial activity against both Gram-positive (*Staphylococcus aureus* and *Bacillus subtilis*) and Gram-negative (*Escherichia coli* and *Pseudomonas aeruginosa*) bacteria, as well as antifungal activity against *Candida albicans* and *Saccharomyces cerevisiae*. Nanoparticles synthesized by incubation at 40 °C for 12 h showed the strongest antimicrobial activity. These findings demonstrate that cultivation conditions of *C. vulgaris* and the synthesis conditions of the nanoparticles significantly influence nanoparticle properties and biological activity, highlighting the potential of microalgae-mediated green synthesis for developing environmentally friendly antimicrobial nanomaterials.

## 1. Introduction

In recent years, the development of nanoscale materials using nanotechnology has gained momentum. Since traditional nanoparticle synthesis methods have disadvantages such as high energy requirements, the use of toxic chemicals, and harsh reaction conditions, there is growing interest in environmentally friendly and sustainable green synthesis methods [1,2]. In green synthesis, biological sources such as fungi, bacteria, algae, and plant extracts reduce metal ions and support nanoparticle formation with the bioactive components they contain. This method enables the production of low-cost, environmentally friendly, and stable nanoparticles, and the resulting nanoparticles have potential for use in various biomedical applications [2,3].

Algae and microalgae rich in polyphenols, flavonoids, and terpenoids are promising biological sources for green synthesis due to their roles in metal reduction and nanoparticle stabilization. Furthermore, since environmental factors such as pH, temperature, and reaction time directly affect the size and morphology of nanoparticles during synthesis, the optimization of these parameters is of great importance [3,4].

Zirconium dioxide (ZrO_2_) is a transition metal oxide nanoparticle notable for its high corrosion resistance, mechanical strength, and thermal and chemical stability. ZrO_2_ nanoparticles can exist in three crystal phases—monoclinic, tetragonal, and cubic—and their phase structure depends on parameters such as temperature, synthesis method, pH, and dopant type. The tetragonal phase plays a significant role in biomedical applications due to its high stability and photocatalytic and heterogeneous catalytic activity [5,6].

Owing to their biocompatibility, chemical stability, high mechanical strength, and excellent wear resistance, ZrO_2_ nanoparticles have attracted considerable attention for various biomedical applications, including implants, dental materials, and antimicrobial applications [7,8]. Due to their surface charges, they can interact with bacterial cell membranes, thereby enhancing biosorption and bioaccumulation and exhibiting antibacterial activity [9]. ZrO_2_ nanoparticles produced via green synthesis, on the other hand, can penetrate the cell membrane, disrupt metabolic processes, and lead to the inactivation of bacterial cells [9].

ZrO_2_ nanoparticles are among the nanomaterials attracting attention not only in biomedical applications but also in antibacterial applications with their superior biocompatibility, high mechanical strength, and low cost. The antibacterial effect of ZrO_2_ nanoparticles is primarily based on electrostatic interactions with the bacterial cell membrane and the mechanism of induced oxidative stress. After encountering bacterial cells, the nanoparticles penetrate the cell, triggering the formation of reactive oxygen species (ROS) and inducing oxidative stress within the cell. Increased ROS levels cause lipid peroxidation, disrupting the integrity of the cell membrane, leading to damage to proteins and genetic material, and ultimately inhibiting cell division, resulting in bacterial death [7,9,10]. The studies in the literature demonstrate that ZrO_2_ nanoparticles are an effective antimicrobial agent against both Gram-positive and Gram-negative bacteria [7,10,11,12,13]. ZrO_2_ nanoparticles produced via green synthesis methods exhibit enhanced physicochemical properties and better biocompatibility due to the coating formed on their surface by biological molecules during synthesis [14,15,16,17,18]. It has been reported that these properties positively contribute to antibacterial efficacy by enhancing the interaction of the nanoparticles with bacterial cells [19,20,21].

*Chlorella vulgaris* (*C. vulgaris*), a species of eukaryotic green microalgae, has attracted significant attention in recent years due to its biotechnological potential. Its rapid growth capacity, high tolerance to environmental conditions, rich chemical composition [22,23,24], and various bioactive components make this microalga stand out in biotechnological applications. Studies in the literature indicate that metabolites produced by *C. vulgaris* possess various biological activities, such as antioxidant [25,26,27,28], immunomodulatory [29,30], and anticancer [31,32,33] properties. Furthermore, the metabolites and pigments present in its composition enable it to function as a reducing and stabilizing agent in the green synthesis of nanoparticles. Therefore, *C. vulgaris* is considered an effective and sustainable biological resource for the environmentally friendly synthesis of nanoparticles [34,35,36].

Antimicrobial resistance ranks among the top global health challenges. Although many antimicrobial agents are widely used in the treatment of infections, the increasing prevalence of resistant microorganisms is limiting the effectiveness and use of these agents. Therefore, research aimed at developing safe, effective, and novel antimicrobial agents is gaining importance. In this context, *C. vulgaris* is one of the microalgae that has attracted attention due to the bioactive compounds it contains. Studies in the literature have demonstrated that the phenolic compounds of *C. vulgaris* exhibit antibacterial activity against both Gram-positive and Gram-negative human pathogens [27,37,38,39]. However, it has been reported that the phenolic compound content of microalgae is significantly dependent on cultivation conditions, and this can directly affect their biological activity [37,38,39,40].

Recent studies have demonstrated the potential of zirconia-based nanomaterials as components of antimicrobial and biomedical systems. Khan et al. reported that surface functionalization of ZrO_2_ nanoparticles with glutamic acid enhanced their antimicrobial activity, highlighting the importance of nanoparticle surface chemistry in their biological interactions [41]. Similarly, Aati et al. demonstrated that incorporation of functionalized ZrO_2_ nanoparticles into 3D-printed dental resins significantly improved antimicrobial activity and reduced biofilm formation without compromising biocompatibility [42], while Chowdhury et al. investigated green-synthesized zirconium nanoparticles for potential dental implant applications [43]. These findings support the potential of ZrO_2_-based nanomaterials for biomedical and antimicrobial applications and provide a broader context for investigating biologically synthesized ZrO_2_ nanoparticles.

In this study, the aim is to synthesize ZrO_2_ nanoparticles using the green synthesis method via *C. vulgaris*, to perform their physicochemical characterization, and to evaluate their antibacterial and antifungal activities. Furthermore, the study aimed to investigate the effect of environmental conditions on the role of bioactive compounds present in *C. vulgaris*, which act as reducing and stabilizing agents in nanoparticle synthesis, on the antimicrobial properties of the resulting ZrO_2_ nanoparticles.

## 2. Results and Discussion

### 2.1. Total Phenolic Content, Total Flavonoid Content and Antioxidant Capacity of C. vulgaris Extracts

In this study, *C. vulgaris* microalgae were cultured at 3 different temperatures and 2 different light period settings. Later, the dry weight obtained after a 14-day cultivation period and the total phenolic content of the extracts were determined in terms of gallic acid equivalents (GAE) [27,44,45] and are presented in Table 1. The data represent the average of results from three independent experiments.

The TPC results show that the total phenolic content of the samples ranged from 4.50 to 9.62 mg GAE/g DW. Among the *C. vulgaris* extracts incubated under different conditions, the lowest phenolic content was detected in the extract incubated at 30 °C with continuous (24 h) lighting, while the highest phenolic content was detected in the *C. vulgaris* extract incubated at 20 °C with a 12 h light period. However, it was observed that the total phenolic content decreased as the light period interval increased, and similarly, the total phenolic content also decreased as the temperature increased.

The total flavonoid content of *C. vulgaris* extracts was also determined using the aluminum chloride colorimetric method [46], and the results are presented as catechin equivalents (CE) in Table 2. According to the table, the total flavonoid content of *C. vulgaris* extracts ranged from 8.24 to 15.75 mg CE/g DW. It was determined that the highest flavonoid content was found in the *C. vulgaris* extract incubated at 25 °C with a 12 h light period, while the lowest flavonoid content was found in the *C. vulgaris* extract incubated at 30 °C with continuous lighting, i.e., a 24 h light period. While an increase in temperature resulted in a decrease in flavonoid content in the extracts incubated for 24 h of lighting, in the extracts incubated with a 12 h light period, the flavonoid content initially increased when the temperature rose from 20 °C to 25 °C, but decreased again when the temperature reached 30 °C.

The antioxidant capacity of *C. vulgaris* extracts was measured using the DPPH assay [28,47], and the results are presented in Table 3. DPPH inhibition in *C. vulgaris* extracts incubated under different conditions ranged from 39.26% to 53.61%. The highest inhibition among these extracts was observed in the *C. vulgaris* extract incubated at 25 °C with a 12 h light period, while the lowest inhibition was observed in the *C. vulgaris* extract incubated at 30 °C with continuous (24 h) lighting. However, inhibition decreased as the incubation times of the *C. vulgaris* extracts increased. In extracts incubated with 24 h lighting, inhibition decreased with increasing temperature; in extracts incubated with a 12 h light period, inhibition initially increased as the temperature rose from 20 °C to 25 °C, but then decreased again as the temperature rose from 25 °C to 30 °C.

**Figure 1:**
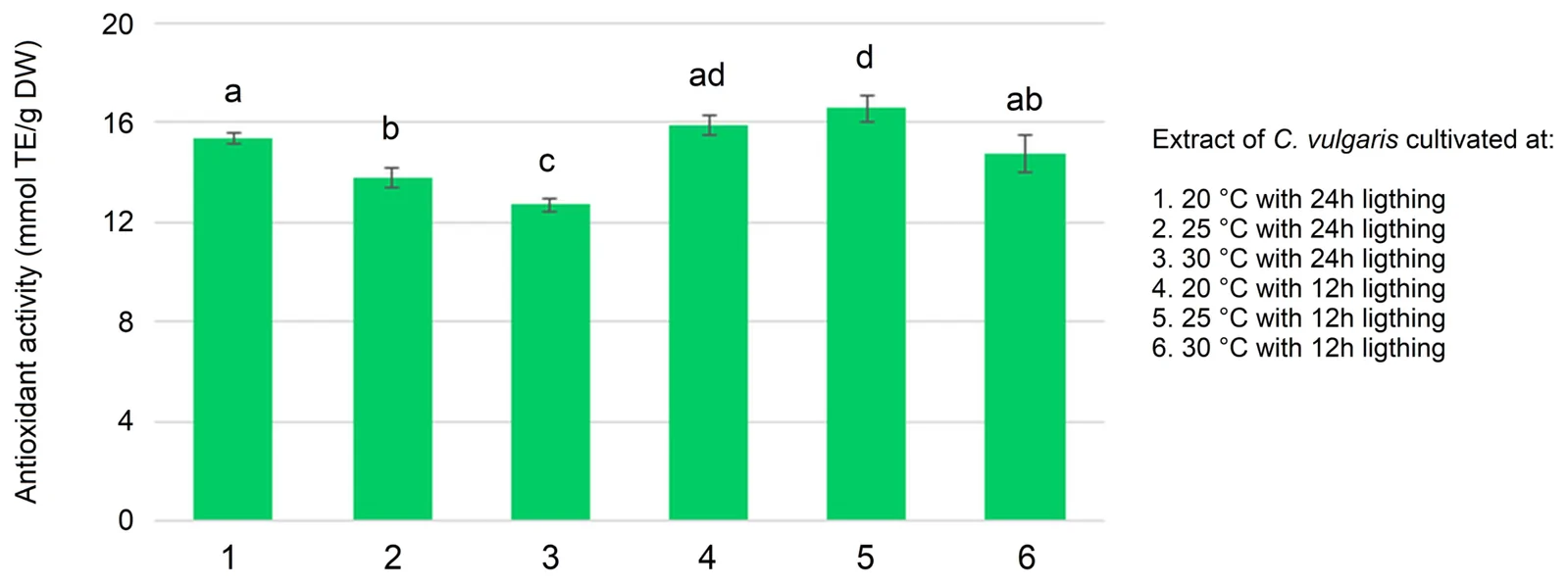
Antioxidant activity of the *C. vulgaris* extracts. Different letters indicate significant differences determined by one-way ANOVA test (*p* < 0.05).

The antioxidant capacity of the *C. vulgaris* extracts prepared in this study is presented in Figure 1 and Supplementary Material Table S1 in terms of Trolox equivalents (TE) [48]. Based on the data in Figure 1, it was observed that the antioxidant activity of the *C. vulgaris* extracts ranged from 12.69 to 16.55 mmol TE/g DW. This analysis revealed that the *C. vulgaris* extract incubated with continuous (24 h) lighting at 30 °C exhibited the lowest antioxidant activity, while the *C. vulgaris* extract incubated with a 12 h light period at 25 °C exhibited the highest antioxidant activity. The findings indicate that the antioxidant activity of the extracts decreased in inverse proportion to the increase in light period. In contrast to the decrease in antioxidant capacity observed with increasing temperature in extracts incubated with continuous (24 h) lighting, in extracts incubated with a 12 h light period, antioxidant activity initially increased with rising temperature but subsequently decreased.

The extract of *C. vulgaris* cultivated at 25 °C with a 12 h light/12 h dark photoperiod outperformed all other conditions tested in this study in terms of TFC activity, DPPH inhibition and antioxidant capacity. The extract obtained at 25 °C under a 12 h light/12 h dark photoperiod showed the highest TFC (15.76 mg CE/g DW), DPPH inhibition (53.61%), and ABTS antioxidant capacity (16.55 mmol TE/g DW), which was statistically significant compared to the other conditions based on the one-way ANOVA test (*p* < 0.05). This extract also demonstrated the second-highest TPC activity, with a relatively high TPC (9.21 mg GAE/g DW). Thus, the extract prepared from *C. vulgaris* cultivation at 25 °C under a 12 h light/12 h dark photoperiod for 14 days was used for the green synthesis of ZrO_2_ nanoparticles.

### 2.2. Green Synthesis of ZrO_2_ Nanoparticles

Green synthesis of ZrO_2_ nanoparticles was successfully achieved using *C. vulgaris* extracts following the method by Sudhakar et al. [49]. Nanoparticle synthesis was tested at two different temperatures (40 °C and 50 °C) and incubation periods (12 h and 24 h) as indicated in Table 4. The obtained nanoparticle powders were subsequently characterized to assess their physicochemical properties and to determine the effect of the different synthesis conditions on nanoparticle formation.

#### 2.2.1. DLS Analysis

DLS was performed to determine the hydrodynamic sizes and polydispersity Index (PDI) values of the ZrO_2_ nanoparticles produced, and the results are presented in Table 4. As a result of the DLS analysis, it was determined that the average hydrodynamic sizes of the ZrO_2_ nanoparticles synthesized using *C. vulgaris* extracts under different conditions ranged from 419 to 570 nm. The smallest average nanoparticle size was observed in the ZrO_2_ nanoparticles synthesized at 50 °C over 12 h, while the largest average size was observed in the nanoparticles synthesized at 50 °C over 24 h.

As part of the DLS analysis, the PDI values of the nanoparticles were also evaluated, and it was determined that the PDI values ranged from 0.27 to 0.44. The highest PDI value was observed in the ZrO_2_ nanoparticles synthesized at 40 °C over 24 h (PDI = 0.44), while the lowest PDI value was observed in the nanoparticles synthesized at 40 °C over 12 h (PDI = 0.27). These findings indicate that the nanoparticle synthesis temperature and duration may influence the hydrodynamic size and PDI value of the synthesized ZrO_2_ nanoparticles.

#### 2.2.2. FTIR Analysis

FTIR analysis was performed to identify the functional groups of the *C. vulgaris* extract and the synthesized nanoparticles used in this study.

**Figure 2:**
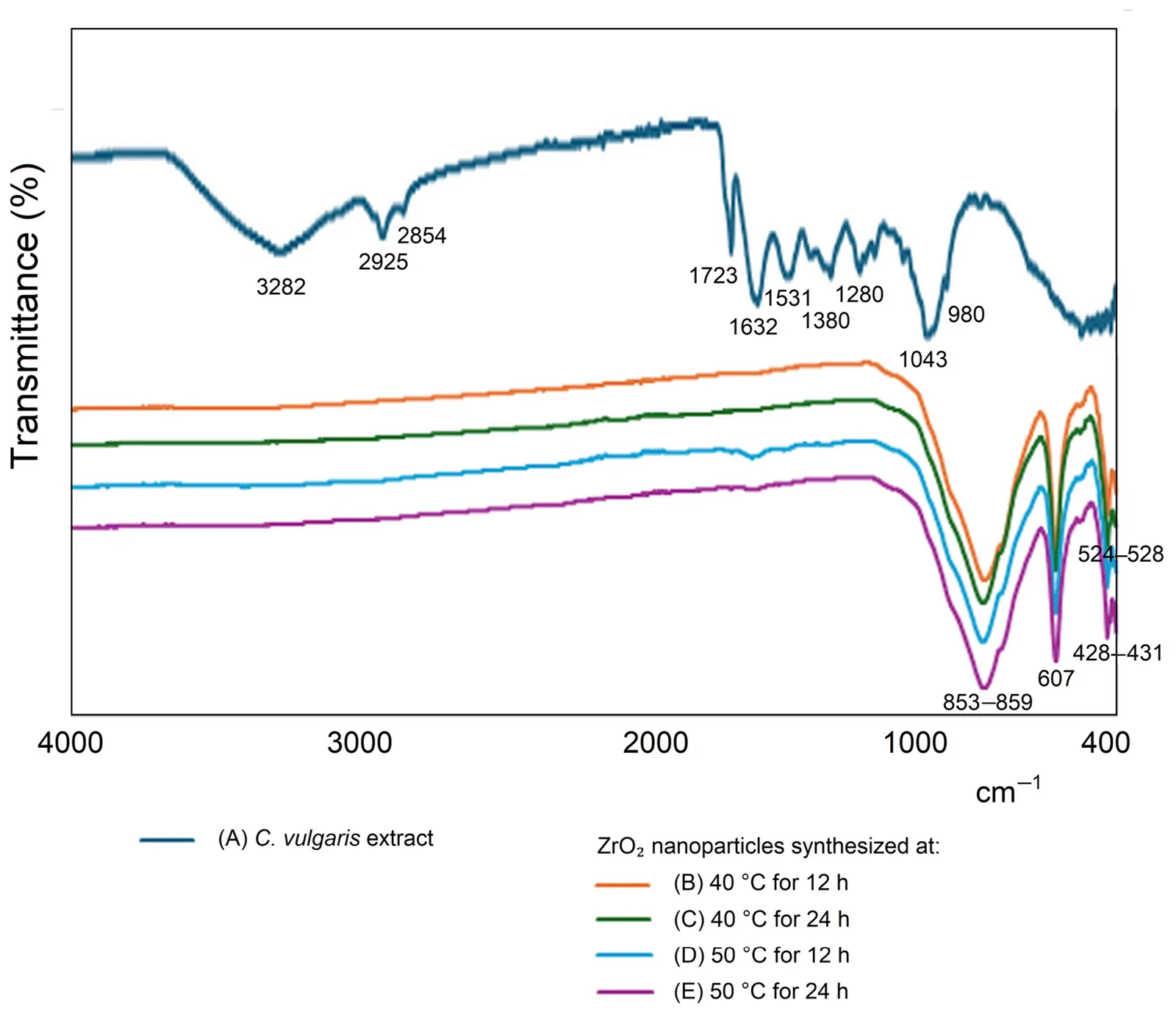
FTIR spectra of (A) *C. vulgaris* extract and ZrO_2_ nanoparticles synthesized using *C. vulgaris* extracts incubated under different conditions: (B) 40 °C for 12 h, (C) 40 °C for 24 h, (D) 50 °C for 12 h, and (E) 50 °C for 24 h.

The FTIR spectrum of the *C. vulgaris* extract is shown in Figure 2(A). The phytochemical screening of the *C. vulgaris* extract indicated the presence of flavonoids, quinones, alkaloids, terpenoids, and glycosides, while the quantitative analyses demonstrated appreciable levels of phenolic and flavonoid compounds (Supplementary Material, Table S3). These phytochemical classes, together with proteins, carbohydrates, lipids, pigments, and other algal metabolites, contain functional groups that may participate in zirconium precursor conversion and stabilization of the resulting nanoparticles. The FTIR spectrum of the *C. vulgaris* extract showed a broad band at 3282 cm^−1^, attributable to O–H/N–H stretching, while the bands at 2925 and 2854 cm^−1^ were associated with aliphatic C–H stretching, and the band at 1723 cm^−1^ with C=O stretching. The bands at 1632 and 1531 cm^−1^ were associated with protein-related amide I and amide II vibrations, supporting the contribution of proteinaceous and other oxygen- and nitrogen-containing biomolecules present in the extract. Such functional groups are consistent with the phytochemical composition of *C. vulgaris* and may act as reducing, coordinating, and/or capping sites during nanoparticle formation, as reported for other *C. vulgaris*-mediated nanoparticle syntheses [35,36]. Following synthesis, the predominance of bands assigned to Zr–O and Zr–O–Zr vibrations, together with changes in the organic-associated bands, suggests that extract-derived biomolecules participated in the formation and stabilization of the ZrO_2_ nanoparticles.

The FTIR spectrum of ZrO_2_ nanoparticles synthesized using *C. vulgaris* extract at 40 °C over 12 h is presented in Figure 2(B). In the spectrum, while the characteristic organic functional group bands identified in the *C. vulgaris* extract were only observed in minuscule amounts, bands associated with metal–oxygen bonds were detected. In particular, the bands observed at 853, 607, 526, 428, and 410 cm^−1^ were attributed to Zr–O and Zr–O–Zr vibrations [50]. Absence of bands in the 1300–1550 cm^−1^ region that would correspond to the nitrate (NO_3_^−^) stretching and bending [51] confirmed full conversion of zirconium nitrate into ZrO_2_ nanoparticles.

Similarly, the FTIR spectrum of ZrO_2_ nanoparticles synthesized using *C. vulgaris* extract by incubation for 24 h at 40 °C (Figure 2(C)) showed distinct absorption bands at 857, 607, 524, and 430 cm^−1^. These bands have been associated with the characteristic vibrations of Zr–O and Zr–O–Zr bonds.

The FTIR spectrum of ZrO_2_ nanoparticles synthesized over 12 h at 50 °C is shown in Figure 2(D). In this spectrum as well, characteristic bands attributed to Zr–O and Zr–O–Zr bonds were observed. However, in the sample synthesized by incubation at 40 °C for 24 h, the band observed at 857 cm^−1^ was found to have shifted to 859 cm^−1^.

In the FTIR spectrum of ZrO_2_ nanoparticles synthesized at 50 °C for 24 h, characteristic absorption bands were observed at 855, 607, 526, 430, 419, and 408 cm^−1^ (Figure 2(E)). These bands are attributed to the vibrations of Zr–O and Zr–O–Zr bonds. When compared to the spectrum of nanoparticles synthesized by a 12 h incubation at 50 °C, it was determined that the band at 607 cm^−1^ retained its position. In contrast, the band at 859 cm^−1^ was observed to shift to 855 cm^−1^.

#### 2.2.3. SEM

**Figure 3:**
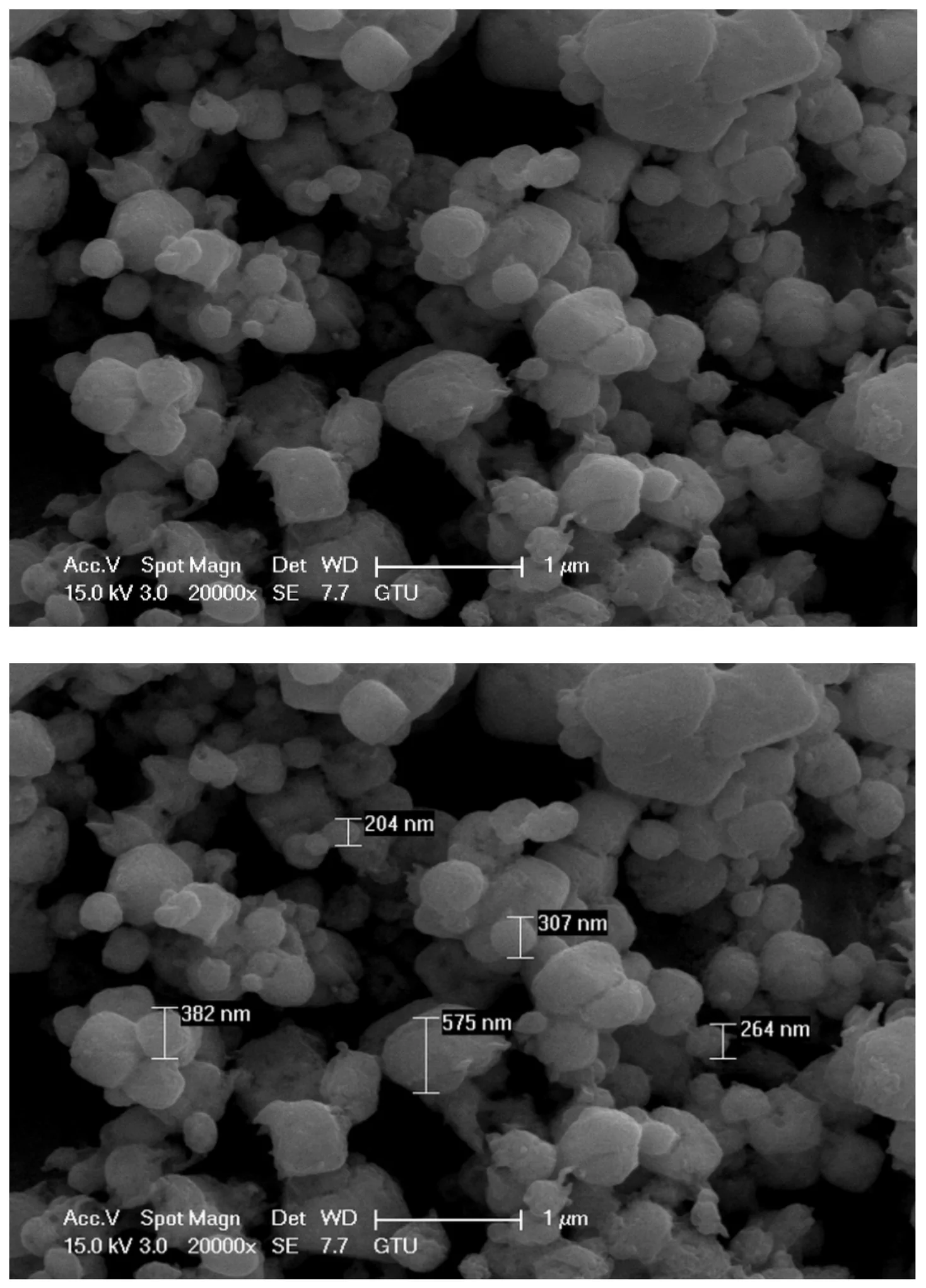
SEM results of ZrO_2_ nanoparticles synthesized at 40 °C over 12 h.

The surface morphology and size characteristics of ZrO_2_ nanoparticles synthesized using *C. vulgaris* extract at 40 °C over 12 h were examined by SEM, and the images are shown in Figure 3. According to the SEM results, the synthesized ZrO_2_ nanoparticles have a spherical morphology, and their surfaces are generally smooth. However, the size distribution of these nanoparticles appears to be heterogeneous. The SEM analysis revealed that the sizes of the synthesized ZrO_2_ nanoparticles vary widely, ranging from 204 nm to 575 nm. These results indicate that the nanoparticle sizes are not homogeneous but vary in size, consistent with the PDI data obtained from DLS. The SEM analysis of the ZrO_2_ nanoparticles synthesized using *C. vulgaris* extract at 40 °C for 24 h is presented for comparison in the Supplementary Material (Figure S1). The nanoparticles synthesized at 40 °C for 24 h exhibited a greater degree of morphological heterogeneity compared with those synthesized at 40 °C for 12 h, which is consistent with the higher PDI value (0.44) observed for the former.

#### 2.2.4. XRD

**Figure 4:**
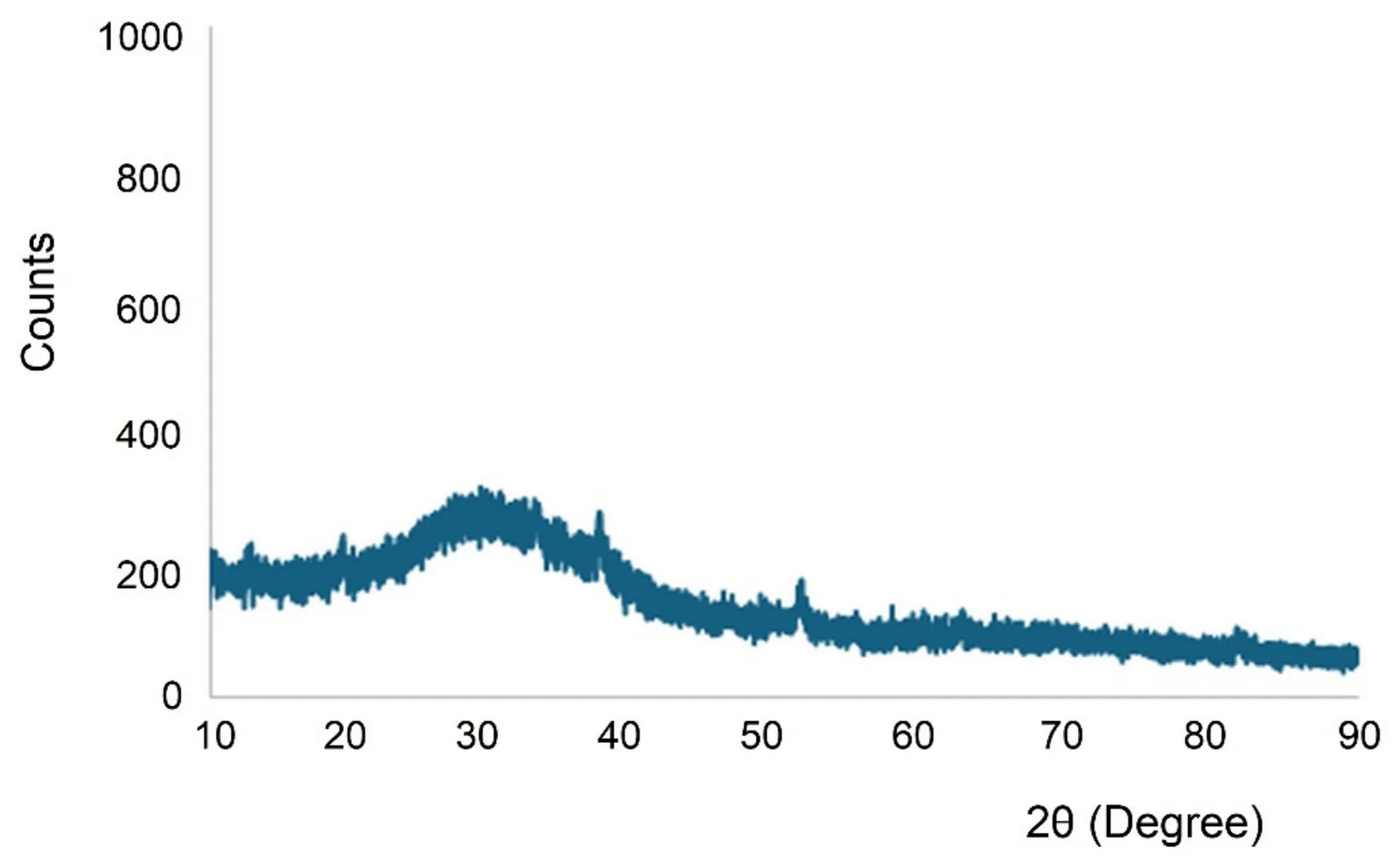
XRD results of ZrO_2_ nanoparticles synthesized at 40 °C over 12 h.

The XRD pattern (Figure 4 and Supplementary Material, Figure S2) exhibited broad, low-intensity diffraction peaks characteristic of a predominantly amorphous structure. Nevertheless, several distinct reflections corresponding to crystalline phases of zirconium dioxide (ZrO_2_) were observed, indicating the formation of a mixed amorphous–crystalline material through the green synthesis process. The coexistence of crystalline and amorphous regions may contribute to the structural stability and surface reactivity of the synthesized nanoparticles [52]. The observed peak broadening is consistent with the heterogeneous morphology observed in the SEM analysis and may be associated with the presence of organic biomolecules derived from the *C. vulgaris* extract acting as surface-capping or stabilizing agents (Figure 2 and Figure 3). Collectively, these findings indicate that the synthesized material is semi-crystalline ZrO_2_, comprising both amorphous and crystalline domains, in agreement with previous reports of biologically mediated zirconia synthesis [19,52,53]. Amorphous or poorly crystalline ZrO_2_ nanoparticles have also been reported following various chemical synthesis procedures, particularly prior to calcination, with broad or poorly defined XRD patterns reflecting a high degree of structural disorder [53,54].

### 2.3. Total Phenolic Content, Total Flavonoid Content and Antioxidant Capacity of ZrO_2_ Nanoparticles

The determination of total phenolic and flavonoid contents in the synthesized nanoparticles was performed to evaluate whether extract-derived phenolic and flavonoid compounds remained associated with the nanoparticles following synthesis. This assessment was particularly relevant because phenolic and flavonoid compounds possess reported antimicrobial properties and may contribute to the biological activity of the synthesized nanoparticles, in addition to the intrinsic antimicrobial effects of ZrO_2_. Therefore, TPC and TFC measurements provide complementary information for interpreting the antimicrobial activity of the green-synthesized ZrO_2_ nanoparticles.

The total phenol content of the ZrO_2_ nanoparticles produced in this study was measured using the GAE method, and the results are presented in Table 5, as the average of results from three independent experiments. The results show that the total phenol content of the ZrO_2_ nanoparticles ranged from 4.82 to 7.18 mg GAE/g DW. The lowest total phenolic content was observed in the ZrO_2_ nanoparticles synthesized by incubation at 50 °C for 24 h, while the highest phenolic content was observed in the ZrO_2_ nanoparticles synthesized at 40 °C over 12 h. These results indicate that an increase in the nanoparticle synthesis time and temperature leads to a decrease in the total phenolic content of the ZrO_2_ nanoparticles. Moreover, the TPC activity of the ZrO_2_ nanoparticles is found to be less than the activity of the *C. vulgaris* extract.

The total flavonoid content of the ZrO_2_ nanoparticles was determined using the aluminum chloride colorimetric method, and the results are presented as catechin equivalents (CE) in Table 6. According to the table, the TFC of ZrO_2_ nanoparticles ranged from 8.06 to 11.74 mg CE/g DW. It was determined that the highest flavonoid content was observed in the ZrO_2_ nanoparticles synthesized at 40 °C for 12 h, while the lowest TFC corresponded to the one incubated at 50 °C for 12 h. Thus, an increase in temperature resulted in a decrease in flavonoid content in the ZrO_2_ nanoparticles. Moreover, the TFC activity of the ZrO_2_ nanoparticles is found to be less than the activity of the *C. vulgaris* extract.

The antioxidant capacity of the synthesized ZrO_2_ nanoparticles was measured using the DPPH assay, and the results are presented in Table 7. As shown in the table, the DPPH inhibition values of ZrO_2_ nanoparticles synthesized under different conditions ranged from 57.78% to 68.21%. The highest DPPH inhibition percentage was observed in the ZrO_2_ nanoparticles synthesized at 40 °C over 12 h, while the lowest DPPH inhibition percentage was observed in the ZrO_2_ nanoparticles synthesized at 50 °C over 12 h. In nanoparticles synthesized at 40 °C, an increase in incubation time reduced DPPH inhibition; conversely, in nanoparticles synthesized at 50 °C, an increase in incubation time resulted in an increase in the DPPH inhibition percentage. However, in nanoparticles synthesized with the same incubation time, an increase in temperature was found to result in a decrease in the DPPH inhibition percentage.

**Figure 5:**
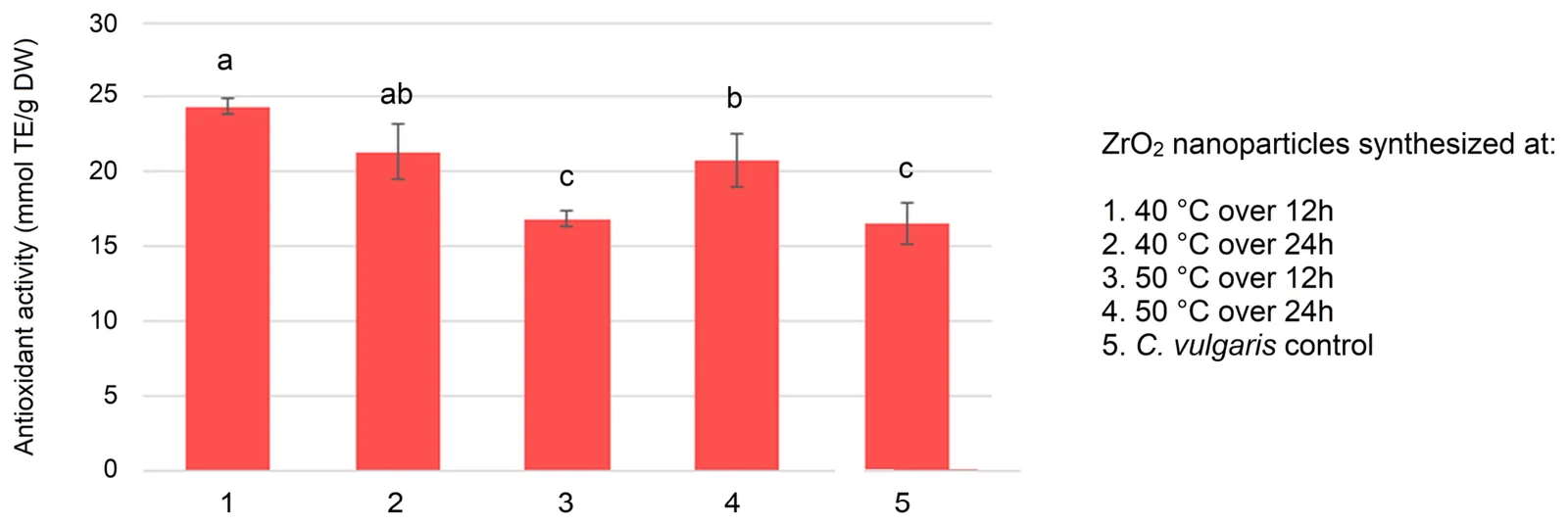
Antioxidant activity of the ZrO_2_ nanoparticles. Different letters indicate significant differences determined by one-way ANOVA test (*p* < 0.05).

The antioxidant capacity of ZrO_2_ nanoparticles produced in this study is presented in Figure 5 and Supplementary Material Table S2 in terms of Trolox equivalents (TE). It was observed that the antioxidant capacities of the ZrO_2_ nanoparticles synthesized by incubation under different conditions ranged from 16.89 to 24.38 mmol TE/g DW. The nanoparticle with the lowest antioxidant capacity was the ZrO_2_ nanoparticle synthesized at 50 °C for 12 h, while the nanoparticle with the highest antioxidant capacity was the ZrO_2_ nanoparticle synthesized at 40 °C over 12 h. While an increase in the incubation time for the synthesis performed at 40 °C led to a decrease in the antioxidant activity of the ZrO_2_ nanoparticles, an increase in the synthesis time for the ones performed at 50 °C led to an increase in the antioxidant activity of the ZrO_2_ nanoparticles. However, an increase in temperature at every incubation time resulted in a decrease in the antioxidant activity of the ZrO_2_ nanoparticles. Moreover, the antioxidant activity of most of the ZrO_2_ nanoparticles synthesized under four different reaction conditions exceeded the antioxidant activity of the *C. vulgaris* extract (Figure 5).

### 2.4. The Antibacterial and Antifungal Activities of ZrO_2_ Nanoparticles

The antibacterial activity of the *C. vulgaris* extract and synthesized ZrO_2_ nanoparticles was evaluated against four bacterial strains: the Gram-negative bacteria *E. coli* and *P. aeruginosa* and the Gram-positive bacteria *S. aureus* and *B. subtilis*. Moreover, antifungal activities were determined against *S. cerevisiae* and *C. albicans.* Different concentrations of ZrO_2_ nanoparticles were added to microbial cultures in a 96-well plate, and the plates were incubated at 37 °C for 24 h for bacterial strains and 30 °C for 48 h for yeasts. Table 8 presents the antimicrobial activities of the *C. vulgaris* extracts and the ZrO_2_ nanoparticles synthesized using these extracts. Ampicillin and fluconazole were used as positive controls for the bacterial and fungal strains, respectively. The ZrO_2_ nanoparticles exhibited antimicrobial activity against all tested microorganisms, although their activity was lower than that of the corresponding positive controls. In general, both the *C. vulgaris* extracts and ZrO_2_ nanoparticles exhibited greater activity against the Gram-negative bacteria *E. coli* and *P. aeruginosa*. Notably, the ZrO_2_ nanoparticles synthesized at 40 °C for 12 h exhibited stronger antibacterial and antifungal activity than those synthesized at 40 °C for 24 h, suggesting that the synthesis conditions influenced the biological performance of the resulting nanoparticles.

## 3. Materials and Methods

### 3.1. Materials

*C. vulgaris* microalgae was purchased from Algaemade (Pataias, Portugal) (https://www.algbe.com), and BG-11 culture medium was obtained from Sisco Research Laboratories (Mumbai, India). All chemicals used in the content analysis of the microalgae extracts were obtained from Merck (Darmstadt, Germany) or Sigma-Aldrich (St. Louis, MO, USA).

### 3.2. Methods

#### 3.2.1. Cultivation of *C. vulgaris* Microalgae

In this study, *C. vulgaris* was cultured in standard BG-11 medium, which contains 1.5 g NaNO_3_, 31.4 mg K_2_HPO_4_, 36 mg MgSO_4_, 36.7 mg CaCl_2_, 20 mg Na_2_CO_3_, 1 mg Na_2_EDTA, 5.6 mg citric acid, and 6 mg ferric ammonium citrate per liter. The prepared culture medium was sterilized by autoclaving at 121 °C for 30 min without pH adjustment.

Cultures were grown in cylindrical containers with a volume of 100 mL of culturing capacity. During microalgae production, the culture medium was aerated at a constant rate of 0.5 L/min per liter of volume. The samples 1–3 were grown under continuous illumination conditions with a light intensity of 100 µE/m^2^·s (measured by a quantum sensor, Licor LI193 SA, LI-COR Biosciences (Lincoln, NE, USA)) by cool-white fluorescent lamps, whereas a 12 h:12 h light:dark period was applied for samples 4–6. The temperature was kept constant according to the values specified in Table 1. Microalgae cultivation was conducted in a batch system under these conditions over a 14-day period. After 14-day cultivation, 50 mL of microalgae culture was centrifuged at 1000× *g* for 15 min, and the supernatant was removed. After carefully washing the cells with dH_2_O, the suspension was centrifuged again, and the settled cells were dried at 60 °C and weighed.

#### 3.2.2. Determination of Total Phenolic Compounds

The total phenolic content (TPC) of the *C. vulgaris* extracts and ZrO_2_ nanoparticles was quantified using the Folin–Ciocalteu assay according to the procedure reported by previous studies [27,44,45] and the ISO standard ISO 14502-1:2005 [45], with minor modifications. Initially, each extract was prepared at a concentration of 1 mg/mL using an ethanol solution (80:20, *v*/*v*). For the colorimetric reaction, 0.5 mL of the diluted sample was combined with 2.5 mL of Folin–Ciocalteu reagent prepared in distilled water. Subsequently, 2.0 mL of 0.4 mol/L sodium carbonate solution was added, and the reaction mixtures were allowed to stand in the dark at room temperature for 2 h to ensure complete color development. The absorbance was recorded at 765 nm using a UV–Vis spectrophotometer (PerkinElmer Lambda 35, Shelton, CT, USA), while the corresponding solvent mixture served as the blank. Gallic acid solutions prepared in ethanol (80:20, *v*/*v*) were used to generate the calibration curve (R^2^ = 0.999). The results are averages of three independent measurements that were calculated from this standard curve and expressed as milligrams of gallic acid equivalents per gram of dry weight (mg GAE/g DW).

#### 3.2.3. Determination of Total Flavonoids

The total flavonoid content (TFC) of the *C. vulgaris* extracts and ZrO_2_ nanoparticles was determined by the aluminum chloride colorimetric method as described previously [46]. *C. vulgaris* extracts were prepared at a final concentration of 1 mg/mL using an ethanol solution (80:20, *v*/*v*). An aliquot (1.0 mL) of each prepared solution was transferred into a test tube and mixed with 0.3 mL of 0.70 mol/L sodium nitrite solution. After incubation for 5 min at room temperature, 0.3 mL of 0.75 mol/L aluminum chloride solution was added. The reaction mixture was allowed to stand for an additional 6 min, followed by the absorbance of the resulting flavonoid–aluminum complexes being measured immediately at 510 nm using ethanol (80:20, *v*/*v*) as the blank. A calibration curve was established using catechin standard solutions prepared in ethanol (80:20, *v*/*v*) (R^2^ = 0.998). The total flavonoid content was calculated as the average of three independent measurements using the standard curve and expressed as milligrams of catechin equivalents per gram of dry weight (mg CE/g DW).

#### 3.2.4. Determination of Antioxidant Activity

The antioxidant activity of the *C. vulgaris* extracts and ZrO_2_ nanoparticles was evaluated using the 2,2-diphenyl-1-picrylhydrazyl (DPPH) free radical scavenging assay according to the method described earlier [28,47]. For each analysis, the reaction mixture consisted of 0.5 mL of the *C. vulgaris* extract prepared at a concentration of 50 mg/L in ethanol, 3.0 mL of ethanol (80:20, *v*/*v*), and 0.3 mL of 0.5 mmol/L DPPH solution prepared in the same solvent system. The reaction mixtures were incubated in the dark at room temperature for 30 min to allow the reduction of DPPH radicals, resulting in a color change from violet to pale yellow. After incubation, the absorbance was measured at 517 nm using a UV–Vis spectrophotometer, with ethanol (80:20, *v*/*v*) serving as the reference. A control solution containing 3.0 mL of ethanol (80:20, *v*/*v*) and 0.3 mL of the DPPH solution, without the addition of *C. vulgaris* extract, was prepared under identical conditions and incubated for 30 min in the dark. Its absorbance at 517 nm was recorded and used as the control value (A_0_). The antioxidant activity was expressed as the percentage of DPPH radical inhibition (% inhibition), calculated according to Equation (1), where A_0_ represents the absorbance of the control solution, and A_S_ corresponds to the absorbance of the sample after the incubation period.

(1) $$\%\;DPPH\;inhibition=\frac{A_{0}-A_{S}}{A_{0}}\times 100$$

The ABTS radical cation (ABTS•^+^) scavenging activity of the *C. vulgaris* extracts and ZrO_2_ nanoparticles was determined according to the method described earlier [48]. The ABTS•^+^ stock solution was prepared by combining 5.0 mL of 7 mmol/L aqueous ABTS solution with 88 μL of 140 mmol/L potassium persulfate solution, corresponding to a final potassium persulfate concentration of 2.45 mmol/L. The mixture was incubated at 25 °C in the dark for 16 h to allow complete generation of the ABTS radical cation, indicated by the development of a dark blue color. Prior to analysis, the ABTS•^+^ stock solution was diluted with an ethanol mixture (80:20, *v*/*v*) until an absorbance of 0.70 ± 0.02 was obtained at 734 nm. For the assay, 3.0 mL of the diluted ABTS•^+^ solution was mixed with appropriate volumes of ethanolic *C. vulgaris* extracts to obtain final sample concentrations of 10 μmol/L. The reaction mixtures were incubated at room temperature for 6 min, after which the absorbance was recorded at 734 nm using ethanol (80:20, *v*/*v*) as the blank. Trolox was used as the reference antioxidant for calibration. A standard calibration curve was prepared using Trolox solutions (R^2^ = 0.997), and the antioxidant capacity of the *C. vulgaris* extracts was calculated from this curve. The results were the averages of three independent measurements and expressed as Trolox equivalent antioxidant capacity (TEAC), reported as millimoles of Trolox equivalents (mmol TE) per gram of dry weight (mmol TE/g DW).

#### 3.2.5. Preparation of the *C. vulgaris* Extract

The *C. vulgaris* extract to be used in this study was prepared by adding 250 mg of *C. vulgaris* to 50 mL of distilled water (dH_2_O). The dispersed mixture was incubated at 60 °C for 1 h. The extracts were then cooled to room temperature and filtered through Whatman filter paper. The extracts were stored in the dark at +4 °C until further processing.

#### 3.2.6. Nanoparticle Synthesis

In this study, the nanoparticle synthesis was based on the study by Sudhakar et al. with minor changes [49]. For nanoparticle synthesis, four different conditions were tested. For this purpose, 10 mL *C. vulgaris* extract was mixed with 90 mL of 10 mM zirconium nitrate solution and incubated at two different temperatures (40 °C and 50 °C) and incubation periods (12 h and 24 h) as indicated in Table 5 by stirring at 200 rpm. The resulting homogeneous solution was frozen at −80 °C for 3 h and then placed in a lyophilizer (Teknosem, İstinye University, Istanbul, Turkey) and lyophilized. The obtained ZrO_2_ nanoparticles in powder form were stored at +4 °C until characterization procedures were performed.

#### 3.2.7. Characterization

Various characterization methods were used to determine the structural and morphological properties of the ZrO_2_ nanoparticles synthesized in this study. SEM (SEM-Philips XL 30 SFEG, Philips Electron Optics, Eindhoven, The Netherlands) was performed to obtain information about the morphology of the nanoparticles [52]. Additionally, DLS (ZEN3600, Malvern Panalytical, Malvern, UK) was performed to determine the hydrodynamic size and polydispersity index of the nanoparticles [52]. X-Ray Diffraction (XRD, Bruker D-8 Advance, Bruker, Karlsruhe, Germany) was conducted to analyze the crystallinity of nanoparticles [52]. FTIR (JASCO4600, Jasco Inc., Easton, MD, USA) was conducted to identify the functional groups and *C. vulgaris* residues present in the structure of the synthesized nanoparticles [50].

#### 3.2.8. Antimicrobial Activity Test Using the MIC Method

The antibacterial and antifungal activity of the synthesized ZrO_2_ nanoparticles and extracts was tested in 24-well microtiter plates by the microwell dilution method as summarized before [55,56]. Bacteria cells (Gram-negative *Escherichia coli* (ATCC^®^ 25922™) and *Pseudomonas aeruginosa* (ATCC^®^ 27853™), and Gram-positive *Staphylococcus aureus* (ATCC^®^ 25923™) and *Bacillus subtilis* (ATCC^®^ 6633™)) were cultured overnight at 37 °C in Lysogeny Broth (LB) and were suspended in LB to give a final density of 1 × 10^6^ CFU/mL. The algal extracts and nanoparticles were put in a 24-well microtiter plate by the serial dilution method. Suspensions of cells without any extract or nanoparticle were tested as negative controls. Ampicillin was used as the positive control for the bacterial strains, and fluconazole was used as the positive control for the fungal strains. The 24-well microtiter plates were incubated to check the viability of the cells for 24 h at 37 °C for bacterial strains and 30 °C for 48 h for yeasts. The antimicrobial tests were reported based on three independent repeats.

#### 3.2.9. Statistical Analyses

Total phenolic content, total flavonoid content, antioxidant capacity, antibacterial and antifungal activity tests were performed at least three times to decrease experimental errors. Total phenolic content, total flavonoid content, and antioxidant capacity tests were reported as the average of three independent measurements, and standard deviations were given. A one-way ANOVA test was applied to the test results of *Chlorella vulgaris* extracts to identify the statistically significantly outperformed and thus the most suitable extract for the green synthesis of zirconium dioxide (ZrO_2_) nanoparticles.

## 4. Conclusions

**Figure 6:**
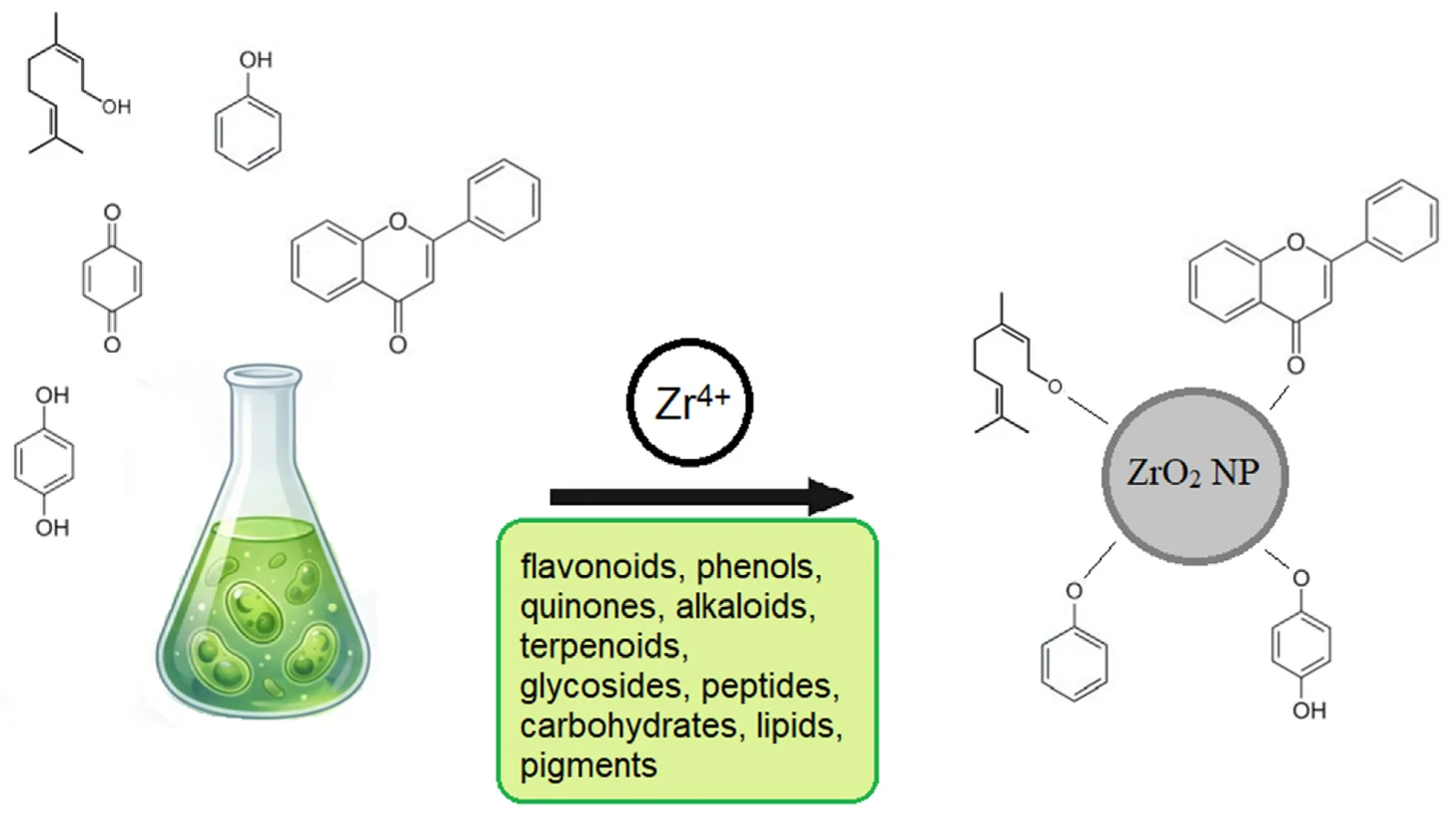
Synthesis and stabilization of ZrO_2_ nanoparticles by bioactive compounds from *C. vulgaris* extract.

This study demonstrates that *Chlorella vulgaris* is an effective biological resource for the green synthesis of ZrO_2_ nanoparticles with promising antioxidant and antimicrobial properties. The phytochemical composition of the *C. vulgaris* extract was evaluated using tests previously described in the literature (Supplementary Material, Table S3). The results indicated that the extract contained flavonoids, quinones, alkaloids, terpenoids, and glycosides. Phenolic and flavonoid compounds, together with proteins, peptides, carbohydrates, lipids, pigments, and other algal metabolites, may contribute to interactions between zirconium species and the biological matrix (Figure 6). Functional groups such as hydroxyl, carbonyl, carboxyl, and amino groups can coordinate with zirconium species and may facilitate precursor conversion, nucleation, and particle growth. These biomolecules may also remain associated with the nanoparticle surface and contribute to stabilization by limiting uncontrolled particle aggregation. This interpretation is consistent with the phytochemical analyses and FTIR results, which showed bands corresponding to O–H/N–H, C–H, C=O, and protein-associated amide groups in the algal extract, whereas the nanoparticle spectra were dominated by metal–oxygen vibrations.

The biochemical composition of the algal extracts was strongly influenced by the cultivation and incubation conditions, which subsequently affected nanoparticle formation and biological performance. Extracts prepared under milder cultivation conditions exhibited higher phenolic and flavonoid contents and antioxidant capacity, resulting in nanoparticles with enhanced antioxidant and antimicrobial properties. Characterization by DLS, FTIR, XRD, and SEM provided complementary evidence supporting the formation of predominantly spherical, semi-crystalline ZrO_2_ nanoparticles with characteristic zirconium–oxygen vibrations and nanoscale-to-submicron particle dimensions. The XRD pattern further indicated the coexistence of amorphous and crystalline domains, suggesting that the green synthesis process produced a semi-crystalline ZrO_2_ material.

The synthesized nanoparticles showed improved antioxidant activity compared with the corresponding algal extracts and effectively inhibited the growth of both Gram-positive and Gram-negative bacteria as well as fungal pathogens, with nanoparticles synthesized at 40 °C for 12 h exhibiting the highest antimicrobial efficacy. Overall, these findings indicate that optimization of *C. vulgaris* cultivation and nanoparticle synthesis conditions represents an effective strategy for tailoring the physicochemical and biological properties of green-synthesized ZrO_2_ nanoparticles. The developed nanoparticles show considerable promise as environmentally friendly antimicrobial nanomaterials for future biomedical and pharmaceutical applications, supporting similar recent findings on green synthesis of ZrO_2_ nanoparticles [52,53,57], although further studies investigating their cytotoxicity, biocompatibility, and mechanisms of antimicrobial action are warranted.

## Figures and Tables

**Table 1:** The dry weight and the total phenolic content (TPC) for the *C. vulgaris* cultures.

Sample	Temperature	Light Period	Dry Weight (mg/L)	TPC Activity (mg GAE/g DW)
Sample 1	20 °C	24 h	121 ± 9	6.85 ± 0.45
Sample 2	25 °C	24 h	145 ± 12	5.49 ± 0.53
Sample 3	30 °C	24 h	134 ± 14	4.50 ± 0.36
Sample 4	20 °C	12 h	95 ± 8	9.62 ± 0.27
Sample 5	25 °C	12 h	118 ± 11	9.21 ± 0.72
Sample 6	30 °C	12 h	129 ± 13	7.43 ± 0.67

**Table 2:** The total flavonoid content (TFC) of the *C. vulgaris* extracts.

Sample	TFC Activity (mg CE/g DW)
Sample 1 (20 °C 24 h)	12.62 ± 0.66
Sample 2 (25 °C 24 h)	9.82 ± 0.65
Sample 3 (30 °C 24 h)	8.24 ± 0.62
Sample 4 (20 °C 12 h)	12.40 ± 0.88
Sample 5 (25 °C 12 h)	15.76 ± 1.06
Sample 6 (30 °C 12 h)	12.94 ± 1.59

**Table 3:** % DPPH inhibition by the *C. vulgaris* extracts.

Sample	% DPPH Inhibition
Sample 1 (20 °C 24 h)	49.21 ± 3.87
Sample 2 (25 °C 24 h)	41.44 ± 3.47
Sample 3 (30 °C 24 h)	39.26 ± 5.75
Sample 4 (20 °C 12 h)	52.75 ± 2.96
Sample 5 (25 °C 12 h)	53.61 ± 4.68
Sample 6 (30 °C 12 h)	44.91 ± 2.68

**Table 4:** Hydrodynamic Size and PDI Values of Synthesized ZrO_2_ Nanoparticles.

Nanoparticle	Conditions of Nanoparticle Synthesis	Mean Hydrodynamic Size	PDI Value
Sample 1	12 h @40 °C	467 nm	0.27
Sample 2	24 h @40 °C	455 nm	0.44
Sample 3	12 h @50 °C	419 nm	0.36
Sample 4	24 h @50 °C	570 nm	0.38

**Table 5:** The total phenolic content (TPC) of the ZrO_2_ nanoparticles.

Sample	Activity (mg GAE/g DW)
Sample 1 (40 °C 12 h)	7.18 ± 0.70
Sample 2 (40 °C 24 h)	5.33 ± 0.50
Sample 3 (50 °C 12 h)	4.92 ± 0.17
Sample 4 (50 °C 24 h)	4.82 ± 0.74
Sample 5 (*C. vulgaris* control)	9.21 ± 0.72

**Table 6:** The total flavonoid content (TFC) of the ZrO_2_ nanoparticles.

Sample	Activity (mg CE/g DW)
Sample 1 (40 °C 12 h)	11.74 ± 1.47
Sample 2 (40 °C 24 h)	11.16 ± 2.07
Sample 3 (50 °C 12 h)	8.06 ± 0.76
Sample 4 (50 °C 24 h)	9.23 ± 0.65
Sample 5 (*C. vulgaris* control)	15.76 ± 1.06

**Table 7:** % DPPH inhibition by the ZrO_2_ nanoparticles.

Sample	% DPPH Inhibition
Sample 1 (40 °C 12 h)	68.21 ± 5.08
Sample 2 (40 °C 24 h)	65.14 ± 3.25
Sample 3 (50 °C 12 h)	57.78 ± 6.57
Sample 4 (50 °C 24 h)	59.09 ± 3.56
Sample 5 (*C. vulgaris* control)	53.61 ± 4.71

**Table 8:** Antimicrobial activity (MIC) of the *C. vulgaris* extract and ZrO_2_ nanoparticles.

	*C. vulgaris* Extract	ZrO_2_ NP (40 °C 12 h)	ZrO_2_ NP (40 °C 24 h)	Ampicillin	Fluconazole
*E. coli*	10 µg/mL	125 µg/mL	250 µg/mL	10 µg/mL	-
*P. aeruginosa*	10 µg/mL	125 µg/mL	250 µg/mL	40 µg/mL	-
*S. aureus*	20 µg/mL	125 µg/mL	125 µg/mL	10 µg/mL	-
*B. subtilis*	20 µg/mL	250 µg/mL	250 µg/mL	10 µg/mL	-
*S. cerevisiae*	40 µg/mL	250 µg/mL	500 µg/mL	-	10 µg/mL
*C. albicans*	20 µg/mL	250 µg/mL	500 µg/mL	-	10 µg/mL

## Data Availability

The original contributions presented in this study are included in the article/Supplementary Materials. Further inquiries can be directed to the corresponding author.

## Supplementary Materials

The following supporting information can be downloaded at: https://www.mdpi.com/article/10.3390/ijms27177803/s1. References [25,58] are cited in the Supplementary Materials.

## Abbreviations

ABTS, 2,2′-azinobis(3-ethylbenzothiazoline-6-sulfonic acid); DLS, dynamic light scattering; DPPH, 2,2-diphenyl-1-picrylhydrazyl; FTIR, Fourier-transform infrared spectroscopy; GAE, gallic acid equivalents; PDI, polydispersity index; SEM, scanning electron microscopy; TEAC, Trolox equivalent antioxidant capacity; TFC, total flavonoid content; TPC, total phenolic content; ZrO_2_, zirconium dioxide.
