# Supplementary material for: Green Synthesis of Zirconium Dioxide Nanoparticles Using Chlorella vulgaris Extracts with High Antibacterial and Antifungal Properties
Source: Int J Mol Sci. 2026 Aug 31;27(17):7803. doi: 10.3390/ijms27177803 (PMC13565946; doi:10.3390/ijms27177803)
Supplement: Supplementary file 1 [file ijms-27-07803-s001.zip › ijms-4508274-supplementary.pdf]

Supplementary Material

Green Synthesis of Zirconium Dioxide Nanoparticles Using *Chlorella vulgaris* Extracts with High Antibacterial and Antifungal Properties

Beyzanur Erk [1] and Bengü Ergüden [2] *

[1] Department of Biomedical Engineering, İstinye University, İstanbul, Türkiye; beyzanur.erk@istinye.edu.tr
[2] Department of Bioengineering, Gebze Technical University, Kocaeli, Türkiye; b.sezen@gtu.edu.tr

Table S1. Antioxidant activity of the *C. vulgaris* extracts.

Sample	Activity (mmol TE/g DW)
Sample 1 (20 °C 24h)	15.33 ± 0.23
Sample 2 (25 °C 24h)	13.77 ± 0.38
Sample 3 (30 °C 24h)	12.69 ± 0.28
Sample 4 (20 °C 12h)	15.86 ± 0.42
Sample 5 (25 °C 12h)	16.55 ± 0.51
Sample 6 (30 °C 12h)	14.72 ± 0.77

Table S2. Antioxidant activity of the ZrO_2 nanoparticles.

Sample	Activity (mmol TE/g DW)
Sample 1 (40 °C 12h)	24.38 ± 0.50
Sample 2 (40 °C 24h)	21.37 ± 1.79
Sample 3 (50 °C 12h)	16.89 ± 0.51
Sample 4 (50 °C 24h)	20.80 ± 1.74
Sample 5 (*C. vulgaris* control)	16.55 ± 1.41

SEM analyses of ZrO_2 nanoparticles

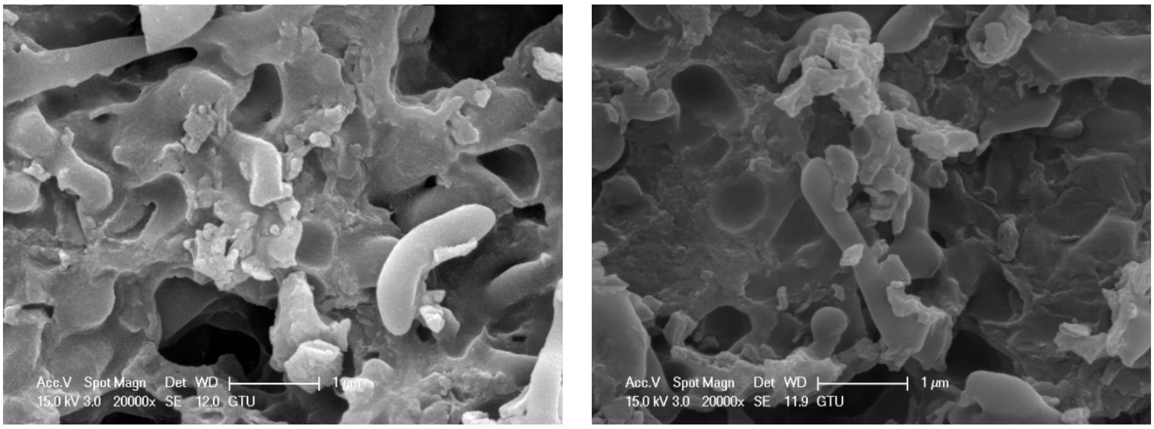

Figure S1. SEM analysis results of ZrO_2 nanoparticles synthesized at 40 °C over 24 hours.

XRD analyses of ZrO_2 nanoparticles

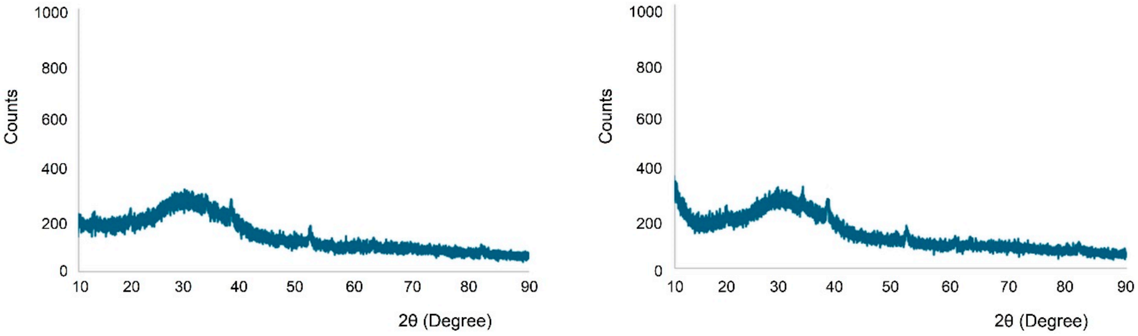

A **B**

Figure S2. XRD analysis results of ZrO_2 nanoparticles; (A) synthesized at 40 °C over 12 hours (B) synthesized at 40 °C over 24 hours.

Phytochemical Tests of Chlorella vulgaris Extract

Following methods were employed for chemical identification of phytochemical components of *Chlorella vulgaris* extract cultured at 25 °C under a 12 h light/12 h dark photoperiod [1,2].

Test for Alkaloids

After adding three milliliters of 1% concentrated HCl to three milliliters of various extracts in a steam bath, Wagner's reagent was added. Alkaloids are indicated by the ensuing precipitate's turbidity.

Test for Tannins

After adding equal amounts of distilled water and various extracts to one another while stirring, a few drops of $FeCl_3$ were added. The presence of tannins was shown by the production of a green precipitate.

Test for Flavonoids

One milliliter of extract was mixed with one milliliter of 2N NaOH solution. When a yellow precipitate formed, the test for flavonoids was considered successful.

Test for Quinone

Mix one milliliter of the plant extract with one milliliter of strong sulfuric acid. A crimson hue indicates the presence of quinones.

Test for Terpenoids

After dissolving two milliliters of the extract in two milliliters of chloroform, the mixture was dried. After that, two milliliters of concentrated sulfuric acid were added, and the mixture was heated for roughly two minutes. Terpenoids are indicated by a grayish hue.

Test for Glycosides

Add 1 ml of glacial acetic acid, 5% ferric chloride, and a few drops of strong sulfuric acid to 2 ml of plant extract. Glycosides are indicated by a greenish-blue hue.

Test for Coumarin

To one milliliter of plant extract, add one milliliter of 10% sodium hydroxide. The presence of coumarins is indicated by a yellow hue.

Test for Steroids

Add a few drops of strong sulfuric acid, one milliliter of plant extract, and the same amount of chloroform. Steroids are represented by a brown ring, while phytosterols are represented by a bluish-green hue.

Anthocyanin and Betacyanin Test

Heat 2 milliliters of plant extract and one milliliter of 2N sodium hydroxide at 100 degrees Celsius for five minutes. Anthocyanins are suggested by a bluish-green hue, whereas betacyanins are indicated by a yellow hue.

Table S3. Results of the Phytochemical Analyses.

**Phytochemicals**	***C. vulgaris* extract**
Alkaloids	+
Tannins	-
Flavonoids	+++
Quinones	++
Terpenoids	+
Glycosides	+
Coumarins	-
Steroids	-
Cyanin	-

References

1. [1] Abdel-Karim, O. H., Gheda, S. F., Ismail, G. A., & Abo-Shady, A. M. (2019). Phytochemical Screening and antioxidant activity of Chlorella vulgaris. *Delta Journal of Science*, *41*(1), 79-91.
2. [2] Andima, M., Bernard, S., Owor, R., and Edward, A. (2014). Preliminary qualitative analysis of phytochemical constituents of the Endemic Aloe tororoana Raynolds in Tororo, Eastern Uganda. Global Advanced Research Journal of Agricultural Science, 3(3), 2315-5094.
